# Baseline Characteristics Associated with Good Collateral Status Using Hypoperfusion Index as an Outcome

**DOI:** 10.3390/tomography8040159

**Published:** 2022-07-25

**Authors:** Omar Hamam, Tushar Garg, Omar Elmandouh, Richard Wang, Alperen Aslan, Amara Ahmed, Abdallah Moustafa, Vivek Yedavalli

**Affiliations:** 1 Department of Radiology and Radiological Sciences, Division of Neuroradiology, Johns Hopkins School of Medicine, Baltimore, MD 21287, USA; ohamam1@jhmi.edu (O.H.); tgarg3@jhmi.edu (T.G.); rwang93@jhmi.edu (R.W.); aslan.alperenmd@gmail.com (A.A.); 2 Department of Radiology, Mayo Clinic College of Medicine, Jacksonville, FL 32224, USA; omar.elmandouh@mayo.edu; 3 School of Medicine, Florida State University, 1115 West Call Street, Tallahassee, FL 32306, USA; aahmed4298@gmail.com; 4 School of Arts and Sciences, Rutgers University-Newark, 360 Dr. Martin Luther King Jr. Blvd., Hill Hall 325, Newark, NJ 07102, USA; aam394@scarletmail.rutgers.edu

**Keywords:** acute ischemic, hypoperfusion, collaterals status, hypoperfusion index

## Abstract

Up to 30% of ischemic stroke cases are due to large vessel occlusion (LVO), causing significant morbidity. Studies have shown that the collateral circulation of patients with acute ischemic stroke (AIS) secondary to LVO can predict their clinical and radiological outcomes. The aim of this study is to identify baseline patient characteristics that can help predict the collateral status of these patients for improved triage. In this IRB approved retrospective study, consecutive patients presenting with AIS secondary to anterior circulation LVO were identified between September 2019 and August 2021. The baseline patient characteristics, laboratory values, imaging features and outcomes were collected using a manual chart review. From the 181 consecutive patients initially reviewed, 54 were confirmed with a clinical diagnosis of AIS and anterior circulation LVO. In patients with poor collateral status, the body mass index (BMI) was found to be significantly lower compared to those with good collateral status (26.4 ± 5.6 vs. 31.7 ± 12.3; *p* = 0.045). BMI of >35 kg/m^2^ was found to predict the presence of good collateral status. Age was found to be significantly higher (70.5 ± 9.6 vs. 58.9 ± 15.6; *p* = 0.034) in patients with poor collateral status and M1 strokes associated with older age and BMI.

## 1. Introduction

Acute ischemic stroke (AIS) secondary to large vessel occlusion (LVO) comprises approximately 24–38% of all cases [1,2]. Mechanical thrombectomy (MT) has become the standard of care for the treatment of LVOs in anterior circulation strokes within 24 h of symptom onset, leading to improved clinical outcomes [3].

Collateral status (CS), for which digital subtraction angiography is considered the reference standard, has been shown to be an independent predictor for good outcome after MT. Good CS was found to be associated with functional independence, successful reperfusion, as well as both decreased symptomatic intracranial hemorrhage and mortality [4]. Poor CS is associated with increased mortality even after successful recanalization [5]. Collateral circulation is important as it provides the brain tissue with blood supply after the vessel supplying the area has been occluded. Good collateral flow can sustain the ischemic penumbra before reperfusion therapy, thereby minimizing the growth of the ischemic core and leading to less neurological deficit [6].

Recently, there has been an increase in the use of computed tomography perfusion (CTP) with the help of automatic post-processing software. CTP provides non-invasive quantitative and rapid measures to estimate the infarct core and potentially salvageable tissue. With the help of these software, a surrogate of CS has been identified which is called the hypoperfusion index (HI). HI is calculated as the ratio of time-to-maximum (Tmax) concentration of more than 10 s divided by the time-to-maximum concentration of more than 6 s [7]. The Tmax is an artificial perfusion parameter that reflects the time delay between the arrival of contrast bolus into the proximal large arterial circulation and the brain parenchyma, and is calculated through a deconvolution step using an arterial input function [8]. In the DEFUSE 2 cohort study, HI has shown to predict the rate of infarct growth and the functional outcome at 90 days in patients presenting with AIS secondary to LVO [8].

In previous studies, it has been shown that certain baseline patient characteristics and laboratory value changes are associated with increased risk of post-MT complications and worse functional outcomes [9,10]. It has been suggested that the patient characteristics and laboratory values can predict their CS. However, to the best of our knowledge, no study to date has systematically explored these relationships when utilizing HI as an indirect imaging surrogate for CS. The aim of our study was to explore these relationships in patients presenting with AIS secondary to anterior circulation LVO who underwent MT for their stroke management.

## 2. Materials and Methods

### 2.1. Study Population

The study population for this institutional review board (JHU-IRB00269637) approved respective study was consecutive patients with anterior circulation LVO who underwent baseline computed tomography angiography (CTA) and CTP followed by MT for their stroke management from September 2019 to August 2021. Anterior circulation LVO was defined as an occlusion of the intracranial internal carotid artery (ICA), M1 or proximal M2 segments of the middle cerebral artery (MCA).

### 2.2. Technical Parameters

Baseline comprehensive CT imaging was performed at the Johns Hopkins Hospital and Johns Hopkins Bayview Medical Centers using helical scanners on the Siemens Flash and/ or Drive (Siemens Healthineers, Erlangen, Germany). For non-contrast CT: helical mode at 5 mm slice thickness (ST), 120 kVp, 365 mAs, rotation time 1 s, acquisition time 6–8 s, collimation 128 × 0.6 mm, pitch value 0.55, scan direction craniocaudal. For CTP: injection of 50 mL non-ionic iodinated contrast with 30 mL saline flush at 5–6 mL/s with coverage of 70–100 mm at 5 mm ST. CTP parameters: 70 kVp, 200 effective mAs, rotation time 0.25 s, average acquisition time 60 s, collimation 48 × 1.2 mm, pitch value 0.7, 4D range 114 mm × 1.5 s. CTP images were then post-processed using RAPID commercial software (IschemaView, Menlo Park, CA, USA) for generating Tmax maps. For CTA head and neck: non-ionic iodinated contrast with 50–70 mL injected at 5–6 mL/s from the aortic arch through the vertex using a bolus triggered method at 3 mm ST. CTA parameters: 90/150 kVp with an Sn filter, quality reference mAs 180, rotation time 0.25 s, average acquisition time 3–5 s, collimation 128 × 0.6 mm, pitch value 0.7, scan direction craniocaudal.

### 2.3. Data Collection

The baseline and clinical data for each patient was collected with the help of a manual chart review performed by O.M.H. The variables collected for each patient included patient demographics, body mass index (BMI)), admission National Institutes of Health Stroke Scale (NIHSS), laboratory values, such as baseline hemoglobin level (Hb), hematocrit (Hct), white blood cell count (WBC), platelet count, platelet/WBC ratio, sodium concentration, potassium concentration, calcium concentration, random blood glucose level, blood urea nitrogen (BUN) level, creatinine level, blood pressure, heart rate, respiratory rate, blood oxygen level measure with SpO_2_ at admission, time from admission to CT, time from admission to IV tPA administration (if applicable), time from admission to groin puncture (if applicable), and groin puncture to recanalization time (if applicable). ASPECTS scores on noncontrast CT were calculated by a board certified neuroradiologist (V.S.Y.)

The CS was quantified using the HI, which was measured using the RAPID commercial software platform (IschemaView, Menlo Park, CA, USA) after post-processing the CTP images. The HI values were dichotomized into poor CS and good CS. Poor CS was defined as an HI of 0.4 or higher while good CS was an HI of less than 0.4.

### 2.4. Study Outcomes

The primary outcome measure was presence of good CS which was defined as HI of less than 0.4.

### 2.5. Statistical Analysis

The data was collected on a secure desktop using Microsoft Office Excel 2007 (Redmond, WA, USA) and analyzed using IBM SPSS statistics (Version 22.0, Chicago, IL, USA). Continuous variables were expressed using mean and SD or median and interquartile range (IQR) based on the distribution of the variable in question. Normality for all continuous variables was assessed using the Shapiro Wilk test. Quantitative data were compared using the independent *t*-test. Qualitative data were compared using Chi square or Fisher’s exact tests. Univariate analysis was initially applied to examine each of the baseline variables independently. Bonferroni corrections were applied for post-hoc tests as multiple comparisons were made on the same dependent variables to reduce the risk of type 1 errors Table 1. All *p*-values were two sided and the *p* value of <0.05 was considered to be statistically significant, unless multiple comparisons were made for which Bonferroni correction was applied, in such cases a *p* value of <0.017 was considered to be statistically significant.

## 3. Results

A total of 54 patients were included in the study cohort. Out of these, 8 (14.8%) patients had an ICA occlusion, 26 (48.1%) patients had a M1 occlusion, and 20 (37.0%) patients had a proximal M2 occlusion.

Table 1 shows the baseline characteristics of the patient cohort with comparison between patients with ICA, M1 and proximal M2 occlusions. The mean age of the patients was 67.9 ± 13.6 years. Fewer than half (48.1%, 26/54) of the patients had a good CS and collateral perfusion. There was no statistically significant difference in the baseline characteristics of patients with ICA, M1 and proximal M2 occlusions, except that the Hct was significantly higher in patients with proximal M2 strokes compared to those with M1 strokes (M1, 37.0 ± 5.2 vs. proximal M2, 41.3 ± 4.4; *p* = 0.005) and hemorrhagic transformation (HT) within 48 H after MT was significantly higher in patients with ICA occlusions compared to M2 occlusions (ICA, 62.5% (5/8) vs. proximal M2, 15% (3/20); *p* = 0.012).

**Figure 1:**
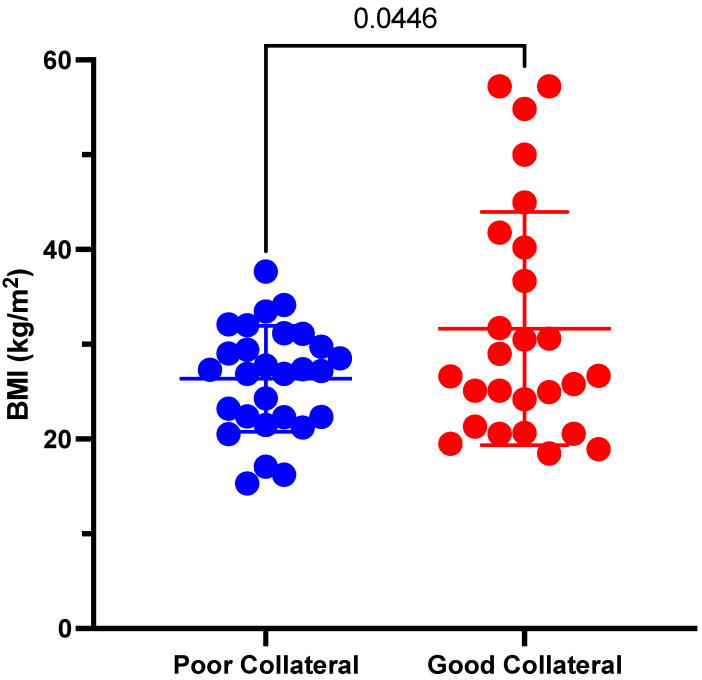
Comparison of BMI in patients with poor vs. good collaterals.

The difference in baseline characteristics of patients with poor and good collateral status is shown in Table 2. The BMI statistically was significantly lower (26.4 ± 5.6 vs. 31.7 ± 12.3; *p* = 0.045) in patients with poor CS compared to patients with good CS (Figure 1).

The comparison in the CS of patients with occlusions in different arterial territories and their relationship to baseline characteristics is shown in Table 3. Age was significantly higher (70.5 ± 9.6 vs. 58.9 ± 15.6; *p* = 0.034) in patients with poor CS and M1 strokes, and BMI was significantly higher (39.7 ± 9.7 vs. 26.8 ± 3.9; *p* = 0.049) in patients with ICA occlusions and good CS. Right sided proximal M2 occlusions were significantly more common in patients with poor CS (63.6% (7/11) vs. 11.1% (1/9); *p* = 0.028) than those with good CS. Diagnostic performance based on vessel subgroup is shown in Table 4. 

## 4. Discussion

In this study, the relationships between baseline patient characteristics and CS were explored, and poor CS was found to be associated with lower BMI. Additionally, the baseline Hct of patients with proximal M2 occlusions was found to be higher than those with M1 occlusions, however, the reason for this association is not clear and needs to be explored in larger studies. The HT rate within 48 H after MT was significantly higher in patients with ICA occlusions compared to M2 occlusions, which differs from prior literature where the rate of HT has been shown to be similar between ICA and MCA occlusions [11]. The reason for this may be due to the much larger volume of territory at risk in ICA occlusions compared to M2 occlusions [12,13].

In patients suffering from AIS, collateral circulation plays an important role in maintaining the blood flow to the tissue at risk of becoming ischemic, and in reducing the risk of hemorrhagic transformation in patients undergoing MT [14]. HI has been shown to be a good surrogate for predicting the CS in patients with acute LVO [15]. Various previous studies have explored the association between patient baseline characteristics and the CS, however variable results have been reported, most likely due to the heterogenous nature of the patient population [16,17,18,19,20,21,22]. Analysis of one of the largest stroke registries, the MR CLEAN Trial and Registry, has shown that older age, male sex, high glucose levels and occlusion of the intracranial internal carotid artery terminus is associated with poor collateral grades as identified on CTA, however, association of various other baseline characteristics, such as body mass index and laboratory values, were not explored in that study [23].

The CS was characterized as poor in 54% of the patients included in this study. This may in part be explained by the older age of the patients included in this cohort. Older age has been associated with progressive loss of number of collaterals and their diameter, along with the increase in arterial tortuosity, all of which leads to an increased resistance in the collateral circulation [24].

Several studies in the literature have shown that higher BMI is associated with reduced cerebral flow and an increased risk of ischemic stroke [25,26,27,28]. However, the outcomes of strokes in patients with obesity have been shown to better than those without due to a multitude of reasons (termed the “obesity paradox”) [27,28,29,30,31]. The obesity paradox can, on a biological level, be explained by the protective effect of soluble tumor necrosis factor-alpha-receptors, which are secreted by the adipose tissue, and bind to the tumor necrosis factor-alpha circulating in the blood [32,33]. Obese patients also have elevated levels of serum lipoproteins and lipids that have been shown to play an important role in blocking the inflammatory cytokine cascade by binding to the liposaccharides in the blood, and this might be responsible for better outcomes in these patients [34,35,36]. Additionally, this study shows that the CS was better in patients with a higher BMI, which could be one of the reasons contributing to better mortality and morbidity outcomes after strokes in patients with obesity.

This study has several limitations to acknowledge. This study was a retrospective analysis at a single center, which can lead to sampling bias, however, we included consecutive patients in the study to minimize this. The data was collected with the help of a retrospective chart review, which can lead to some incorrect recording of data. The study included only patients with acute anterior circulation LVO comprising intracranial ICA, M1, and proximal M2 only. Therefore, these results are not applicable to patients with occlusions of other arterial territories. Patient outcome after MT was not assessed and therefore the results of this study cannot be used to predict the clinical outcomes of patients, although it is again important to note that several prior studies have shown that CS is an important predictive biomarker of patient outcomes, which is the purpose of utilizing CS for the current study. The CS of the patients was assessed utilizing an artificial index for CS estimation in HI. Although HI has been validated as a strong predictor of CS, it still requires further validation in larger prospective cohorts.

## 5. Conclusions

Patients with lower BMI and older age are associated with poor collateral status as predicted by the HI with the help of an automated software. Further investigations are necessary in larger cohorts to validate the results of this study.

## Figures and Tables

**Table 1:** Baseline demographics of the study population and their comparison based on the affected arterial territory.

Variables		All Cases (N = 54)	Arterial Territory			*p*-Value
			ICA (N = 8)	M1 (N = 26)	Proximal M2 (N = 20)	
**Age (years)**		**67.9 ± 13.6**	**74.4 ± 15.7**	64.7 ± 14.0	69.4 ± 11.5	0.175
**Male Sex** **(n%)**		28 (51.9%)	3 (37.5%)	14 (53.8%)	11 (55.0%)	0.811
**Race** **(n%)**	**White/Caucasian**	23 (42.6%)	3 (37.5%)	9 (34.6%)	11 (55.0%)	0.577
	**AfricanAmerican/Black**	30 (55.6%)	5 (62.5%)	16 (61.5%)	9 (45.0%)	
	**Asian**	1 (1.9%)	0 (0.0%)	1 (3.8%)	0 (0.0%)	
**BMI (kg/m^2^)**		28.9 ± 9.7	33.2 ± 9.7	28.3 ± 11.2	27.9 ± 7.4	0.399
**BMI grade**	**<30.0**	36 (66.7%)	5 (62.5%)	19 (73.1%)	12 (60.0%)	0.624
	**≥30.0**	18 (33.3%)	3 (37.5%)	7 (26.9%)	8 (40.0%)	
**Hemoglobin level (gm/dL)**		12.4 ± 2.1	11.9 ± 3.0	11.9 ± 2.0	13.3 ± 1.4	0.061
**Hematocrit (%)**		38.5 ± 5.7	36.4 ± 8.1	37.0 ± 5.2	41.3 ± 4.4	0.019 * p1: 0.799 p2: 0.047 p3: 0.005 *
**WBC count (×10^3^/mL)**		8.7 ± 3.0	8.9 ± 2.4	8.6 ± 3.0	8.7 ± 3.3	0.971
**Platelet count (×10^3^/mL)**		237.3 ± 79.3	223.9 ± 56.0	233.7 ± 70.5	247.4 ± 98.1	0.746
**Platelet/WBC ratio**		29.2 ± 11.3	26.6 ± 8.7	29.1 ± 8.8	30.3 ± 15.0	0.740
**Sodium level (mEq/L)**		139.2 ± 3.2	141.0 ± 4.2	138.3 ± 2.7	139.7 ± 3.1	0.085
**Potassium level (mmol/L)**		4.1 ± 0.5	4.1 ± 0.4	4.1 ± 0.6	4.1 ± 0.4	0.964
**Calcium level (mg/dL)**		8.8 ± 0.5	8.7 ± 0.5	8.9 ± 0.6	8.8 ± 0.5	0.725
**Blood Glucose level (mg/dL)**		135.8 ± 65.1	118.3 ± 10.6	130.4 ± 73.6	149.8 ± 65.5	0.439
**BUN/ creatinine ratio**		18.2 ± 7.8	17.5 ± 7.6	19.8 ± 8.5	16.5 ± 6.8	0.345
**SBP (mmHg)**		148.2 ± 23.7	154.4 ± 21.2	144.2 ± 21.6	150.9 ± 27.3	0.468
**DBP (mmHg)**		82.8 ± 19.9	88.0 ± 23.2	78.4 ± 18.6	86.4 ± 20.0	0.301
**HR (beat/minute)**		80.6 ± 17.8	83.3 ± 20.8	80.9 ± 17.9	79.2 ± 17.2	0.857
**RR (cycle/minute)**		17.6 ± 3.8	17.5 ± 4.3	17.6 ± 3.4	17.6 ± 4.3	0.997
**SpO_2_ (%)**		97.9 ± 2.6	96.6 ± 4.1	98.1 ± 2.2	98.2 ± 2.4	0.329
**NIHSS score**		15.0 ± 7.3	17.8 ± 5.7	15.5 ± 7.2	13.2 ± 7.9	0.307
**Left side improvement** **(n%)**		32 (59.3%)	4 (50.0%)	16 (61.5%)	12 (60.0%)	0.866
**HI**		0.3 ± 0.2	0.3 ± 0.2	0.3 ± 0.2	0.3 ± 0.2	0.990
**Collaterals (n%)**	**Good**	26 (48.1%)	4 (50.0%)	13 (50.0%)	9 (45.0%)	0.933
	**Poor**	28 (51.9%)	4 (50.0%)	13 (50.0%)	11 (55.0%)	
**Hemorrhagic transformation (HT) within 48 H after MT, (n%)**		18 (33.3%)	5 (62.5%)	10 (38.5%)	3 (15.0%)	0.041 * p1: 0.231 p2: 0.012 * p3: 0.080

* Statistically significant (<0.05), p1 is ICA vs. M1, p2 is ICA vs. M2, p3 is M1 vs. M2.

**Table 2:** Comparison in the baseline characteristics according to the collateral status (HI).

Variables		Perfusion		*p*-Value
		Good (N = 26)	Poor (N = 28)	
**Age (years)**		70.7 ± 10.9	65.2 ± 15.3	0.135
**Male Sex** **(n%)**		15 (57.7%)	13 (46.4%)	0.408
**Race** **(n%)**	**White/Caucasian**	14 (53.8%)	9 (32.1%)	0.099
	**African American/Black**	11 (42.3%)	19 (67.9%)	
	**Asian**	1 (3.8%)	0 (0.0%)	
**BMI (kg/m^2^)**		31.7 ± 12.3	26.4 ± 5.6	0.045 *
**BMI grade**	**<30.0 kg/m^2^**	15 (57.7%)	21 (75.0%)	0.178
	**≥30.0 kg/m^2^**	11 (42.3%)	7 (25.0%)	
**Hemoglobin level (gm/dL)**		11.9 ± 2.4	12.9 ± 1.6	0.074
**Hematocrit (%)**		37.4 ± 6.8	39.4 ± 4.4	0.205
**WBC count (×10^3^/mL)**		8.0 ± 2.4	9.3 ± 3.3	0.099
**Platelet count (×10^3^/mL)**		228.2 ± 79.1	245.8 ± 80.0	0.419
**Platelet/WBC ratio**		30.4 ± 12.5	28.1 ± 10.2	0.468
**Sodium level (mEq/L)**		139.9 ± 3.0	138.6 ± 3.3	0.131
**Potassium level (mmol/L)**		4.1 ± 0.4	4.1 ± 0.6	0.817
**Calcium level (mg/dL)**		8.7 ± 0.5	8.9 ± 0.6	0.403
**Blood glucose level (mg/dL)**		127.4 ± 38.1	143.6 ± 82.8	0.368
**BUN/creatinine ratio**		19.0 ± 6.7	17.5 ± 8.8	0.487
**SBP (mmHg)**		150.0 ± 22.6	146.5 ± 25.1	0.597
**DBP (mmHg)**		84.6 ± 20.3	81.1 ± 19.8	0.518
**HR (beat/minute)**		81.0 ± 14.9	80.2 ± 20.4	0.867
**RR (cycle/minute)**		17.0 ± 2.5	18.1 ± 4.7	0.280
**SpO_2_ (%)**		97.8 ± 3.2	98.0 ± 2.0	0.790
**NIHSS score**		13.3 ± 8.1	16.6 ± 6.2	0.102
**ASPECTS score**		9.86 ± 0.14	9.2 ± 0.49	0.696
**Time from door to CT (mins)**		18.28 ± 4.84	14 ± 5.34	0.499
**Time from door to needle (IV TPA) (mins)**		74.28 ± 24.04	50.4 ± 10.67	0.908
**Time from door to groin puncture (MT) (mins)**		167 ± 40.06	122 ± 17.16	0.317
**Time from groin puncture to recanalization (mins)**		34.28 ± 7.86	38 ± 12.14	0.489
**Mechanical Thrombectomy**		26/26 (100%)	26/28 (92.3%)	1
**IV tPA**		8/26 (30.7%)	10/28 (36.3%)	0.758
**Site** **(n%)**	**Right**	8 (30.8%)	14 (50.0%)	0.151
	**Left**	18 (69.2%)	14 (50.0%)	
**Hemorrhagic transformation (HT) within 48 H after MT, (n%)**		9 (34.6%)	9 (32.1%)	0.847

* Statistically significant (<0.05).

**Table 3:** Comparison in the collateral status of patients with occlusions in different arterial territories and their relationship to baseline characteristics.

Variables		ICA			M1 Artery			Proximal M2 Artery		
		Good (N = 4)	Poor (N = 4)	*p*-Value	Good (N = 13)	Poor (N = 13)	*p*-Value	Good (N = 9)	Poor (N = 11)	*p*-Value
**Age (years)**		72.3 ± 16.1	76.5 ± 17.5	0.733	70.5 ± 9.6	58.9 ± 15.6	0.034 *	70.4 ± 11.8	68.5 ± 11.7	0.711
**Male Sex** **(n%)**		1 (25.0%)	2 (50.0%)	0.999	8 (61.5%)	6 (46.2%)	0.431	6 (66.7%)	5 (45.5%)	0.406
**Race** **(n%)**	**White/** **Caucasian**	2 (50.0%)	1 (25.0%)	0.999	6 (46.2%)	3 (23.1%)	0.226	6 (66.7%)	5 (45.5%)	0.406
	**African American/Black**	2 (50.0%)	3 (75.0%)		6 (46.2%)	10 (76.9%)		3 (33.3%)	6 (54.5%)	
	**Asian**	0 (0.0%)	0 (0.0%)		1 (7.7%)	0 (0.0%)		0 (0.0%)	0 (0.0%)	
**BMI (kg/m^2^)**		39.7 ± 9.7	26.8 ± 3.9	0.049 *	31.7 ± 14.6	25.0 ± 4.9	0.138	28.0 ± 8.6	27.8 ± 6.8	0.944
**BMI grade**	**<30.0**	1 (25.0%)	4 (100.0%)	0.143	8 (61.5%)	11 (84.6%)	0.378	6 (66.7%)	6 (54.5%)	0.670
	**≥30.0**	3 (75.0%)	0 (0.0%)		5 (38.5%)	2 (15.4%)		3 (33.3%)	5 (45.5%)	
**Hemoglobin level (gm/dL)**		10.6 ± 3.9	13.1 ± 1.1	0.296	11.4 ± 2.0	12.5 ± 1.9	0.188	13.2 ± 1.7	13.3 ± 1.2	0.796
**Hematocrit (%)**		33.4 ± 10.8	39.3 ± 3.5	0.363	35.6 ± 4.7	38.4 ± 5.4	0.170	41.9 ± 5.8	40.7 ± 3.2	0.563
**WBC count (×10^3^/mL)**		8.7 ± 3.3	9.1 ± 1.7	0.826	8.2 ± 2.9	9.0 ± 3.2	0.505	7.3 ± 0.9	9.8 ± 4.1	0.098
**Platelet count (×10^3^/mL)**		214.0 ± 52.4	233.8 ± 65.7	0.655	228.6 ± 65.7	238.8 ± 77.4	0.722	233.8 ± 109.0	258.5 ± 92.1	0.590
**Platelet/WBC ratio**		26.5 ± 7.3	26.8 ± 11.0	0.968	29.9 ± 8.3	28.3 ± 9.5	0.652	32.8 ± 18.7	28.4 ± 11.6	0.528
**Sodium level (mEq/L)**		143.5 ± 4.0	138.5 ± 3.0	0.094	138.6 ± 2.2	138.1 ± 3.1	0.619	140.2 ± 2.4	139.3 ± 3.6	0.512
**Potassium level (mmol/L)**		4.1 ± 0.4	4.0 ± 0.4	0.675	4.1 ± 0.4	4.1 ± 0.7	0.764	3.9 ± 0.4	4.2 ± 0.4	0.218
**Calcium level (mg/dL)**		8.5 ± 0.5	9.0 ± 0.4	0.165	8.9 ± 0.5	8.8 ± 0.7	0.717	8.6 ± 0.5	8.9 ± 0.5	0.283
**Blood glucose level (mg/dL)**		124.5 ± 5.2	112.0 ± 11.3	0.092	118.7 ± 20.9	142.2 ± 102.7	0.428	141.3 ± 59.4	156.7 ± 72.2	0.614
**BUN/creatinine**		21.0 ± 6.0	14.0 ± 8.0	0.212	18.8 ± 5.8	20.8 ± 10.8	0.576	18.3 ± 8.6	14.9 ± 4.9	0.277
**SBP (mmHg)**		145.0 ± 5.4	163.8 ± 28.0	0.236	150.1 ± 23.8	138.2 ± 18.2	0.167	152.0 ± 26.7	150.0 ± 29.1	0.876
**DBP (mmHg)**		79.5 ± 27.7	96.5 ± 17.2	0.337	80.3 ± 15.7	76.5 ± 21.6	0.616	93.1 ± 22.5	80.8 ± 16.8	0.178
**HR (beat/minute)**		81.0 ± 3.8	85.5 ± 31.3	0.785	83.2 ± 12.6	78.7 ± 22.2	0.535	78.0 ± 20.7	80.1 ± 14.8	0.795
**RR (cycle/minute)**		17.8 ± 4.0	17.3 ± 5.2	0.884	17.0 ± 2.2	18.2 ± 4.3	0.366	16.7 ± 2.4	18.3 ± 5.3	0.387
**SpO_2_ (%)**		96.3 ± 5.7	97.0 ± 2.4	0.816	97.9 ± 2.5	98.3 ± 2.0	0.667	98.3 ± 3.0	98.0 ± 1.9	0.766
**NIHSS score**		16.8 ± 5.0	18.8 ± 7.0	0.658	13.2 ± 8.1	17.6 ± 5.8	0.123	11.9 ± 9.4	14.4 ± 6.4	0.503
**Site** **(n%)**	**Right**	1 (25.0%)	3 (75.0%)	0.486	6 (46.2%)	4 (30.8%)	0.420	1 (11.1%)	7 (63.6%)	0.028 *
	**Left**	3 (75.0%)	1 (25.0%)		7 (53.8%)	9 (69.2%)		8 (88.9%)	4 (36.4%)	
**Hemorrhagic transformation (HT) within 48 H after MT, (n%)**		2 (50.0%)	3 (75.0%)	0.999	6 (46.2%)	4 (30.8%)	0.420	1 (11.1%)	2 (18.2%)	0.362

* Statistically significant (<0.05).

**Table 4:** Diagnostic performance and characteristics of BMI in predicting poor collaterals.

Characteristics	All Cases		ICA		M1		Proximal M2	
	Value	95% CI	Value	95% CI	Value	95% CI	Value	95% CI
**Poor Collaterals from Good Collateral**								
**AUC**	0.560	0.401–0.720	0.813	0.465–1.000	0.550	0.320–0.781	0.465	0.199–0.730
***p*-value**	0.446		0.149		0.663		0.790	
**Cut point**	≤35.0		≤35.0		≤35.0		≤35.0	
**Sensitivity**	96.4%	81.7–99.9%	100%	39.8–100%	100%	75.3–100%	90.9%	58.7–99.8%
**Specificity**	30.8%	14.3–51.8%	75.0%	19.4–99.4%	23.1%	5.0–53.8%	22.2%	2.8–60.0%
**DA**	64.8%	50.6–77.3%	87.5%	47.3–99.7%	61.5%	40.6–79.8%	60.0%	36.1–80.9%
**YI**	27.2%	8.2–46.2%	75.0%	32.6–100%	23.1%	0.2–46.0%	13.1%	18.9–45.2%
**PPV**	60.0%	44.3–74.3%	80.0%	28.4–99.5%	56.5%	34.5–76.8%	58.8%	32.9–81.6%
**NPV**	88.9%	51.8–99.7%	100%	29.2–100%	100%	29.2–100%	66.7%	9.4–99.2%

AUC: Area under the curve; CI: Confidence interval. DA: Diagnostic accuracy. YI: Youden’s Index. PPV: Positive Predictive value. NPV: Negative Predictive value.

## Data Availability

Not applicable.
